# Tumor Immune Microenvironment Components and Checkpoint Molecules in Anaplastic Variant of Diffuse Large B-Cell Lymphoma

**DOI:** 10.3389/fonc.2021.638154

**Published:** 2021-06-16

**Authors:** Tianqi Xu, Jia Chai, Kaijing Wang, Qingge Jia, Yixiong Liu, Yingmei Wang, Junpeng Xu, Kangjie Yu, Danhui Zhao, Jing Ma, Linni Fan, Qingguo Yan, Shuangping Guo, Gang Chen, Qiongrong Chen, Hualiang Xiao, Fang Liu, Chubo Qi, Rong Liang, Mingyang Li, Zhe Wang

**Affiliations:** ^1^ State Key Laboratory of Cancer Biology, Department of Pathology, Xijing Hospital and School of Basic Medicine, Air Force Medical University, Xi’an, China; ^2^ Xi’an International Medical Center, Northwest University, Xi’an, China; ^3^ Department of Pathology, Fujian Cancer Hospital, Fuzhou, China; ^4^ Department of Pathology, Hubei Cancer Hospital, Wuhan, China; ^5^ Department of Pathology, Daping Hospital, Third Military Medical University, Chongqing, China; ^6^ Department of Pathology, The First People’s Hospital of Foshan, Foshan, China; ^7^ Department of Hematology, People’s Liberation Army Centre for Hematologic Disorders, Xijing Hospital, Air Force Medical University, Xi’an, China

**Keywords:** tumor immune microenvironment (TIME), checkpoint molecules, anaplastic variant of diffuse large B-cell lymphoma, PD-L1, prognosis

## Abstract

**Background:**

Anaplastic diffuse large B-cell lymphoma(A-DLBCL) is a rare morphological subtype characterized by the presence of polygonal, bizarre-shaped tumor cells. Our previous research found that A-DLBCL displays many genetic alterations and biological features that differ greatly from those of ordinary DLBCL. However, the status of tumor immune microenvironment components and checkpoint molecules in A-DLBCL remains unclear.

**Methods:**

Thirty A-DLBCL patients were enrolled to study tumor immune microenvironment components and checkpoint molecules and their associations with clinicopathological features and prognosis.

**Results:**

Patients with A-DLBCL presented higher expression of PD-L1 (40% *vs* 10%, P=0.004) than patients with ordinary DLBCL. FISH analysis showed that extra copies of PD-L1 were more frequent in A-DLBCL cases than in ordinary DLBCL cases (23.3% *vs* 4.0%, P=0.001). The numbers of PD-1^+^ TILs (tumor infiltrating lymphocytes) and CD8^+^T cells were significantly lower in A-DLBCL versus ordinary DLBCL. In contrast, the numbers of GATA3^+^ Th2 cells, FOXP3^+^ Tregs and CD33^+^ myeloid-derived suppressor cells (MDSCs) were significantly higher in A-DLBCL than in ordinary DLBCL. The associations between clinicopathological features and tumor immune microenvironment cell frequency were analyzed in A-DLBCL patients. Briefly, the number of PD-1^+^ TILs was lower and the number of CD33^+^ MDSCs was higher in patients with mutated *TP53* compared to those with wild-type *TP53*. The number of FOXP3^+^ Tregs was much lower in patients with a noncomplete response (CR) to chemotherapy than in those with a complete response. The number of CD8^+^ T cells showed a decreasing trend in patients with high International Prognostic Index (IPI) scores and in those with concurrent MYC and BCL2 and/or BCL6 abnormalities. Univariate survival analysis showed that patients with PD-L1^+^, mPD-L1^+^(PD-L1^+^ nonmalignant stromal cells) or mPD-L1^+^ status had a significantly poorer overall survival (OS) than those with PD-L1^-^ status. An increase in the number of CD3^+^ T cells, FOXP3^+^ Treg cells and T-bet^+^ Th1 cells was significantly associated with prolonged OS in patients with A-DLBCL.

**Conclusion:**

Our study suggests that A-DLBCL displays a distinct pattern of tumor immune microenvironment components and checkpoint molecules that distinguish it from ordinary DLBCL. The analysis of tumor immune microenvironment components and checkpoint molecules could help in predicting the prognosis of A-DLBCL patients and determining therapeutic strategies targeting the tumor immune microenvironment.

## Introduction

Diffuse large B-cell lymphoma (DLBCL) is the most common type of lymphoma; it is more prevalent in elderly patients, with a median age in the 70s. However,it also occurs in young adults but rarely occurs in children. Clinically, most patients present with a rapidly growing tumor mass involving one or more lymph nodes and extranodal sites (1, 2). DLBCL displays tremendous heterogeneity in terms of its clinicopathologic and molecular genetic features. DLBCL cases can be subdivided into morphologic variants, molecular and immunophenotypical subgroups, and distinct disease entities.

Anaplastic variant of diffuse large B-cell lymphoma(A-DLBCL) is a rare morphological type, representing approximately 3-4% of all DLBCL cases (1, 2), that is characterized by large or very large, pleomorphic or bizarre-shaped lymphoma cells. Our previous research found that A-DLBCL showed a high frequency of the *TP53* mutation, as well as concurrent abnormalities of *MYC* and *BCL2* and/or *BCL6*, and most cases had a non-GCB immunophenotype. Patients with A-DLBCL follow an aggressive disease course and have poor prognosis (3). In our study on 3 cases of primary CNS A-DLBCL, patients also had *MYC/BCL2* co-expression, and concurrent *MYC* and *BCL2* and/or *BCL6* genetic abnormalities, and constitutive NF-κB pathway activation (4). Our results suggest that A-DLBCL displays many genetic alterations and biological features that differ greatly from ordinary DLBCL.

The PD-1/PD-L1 pathway is an inhibitory immune checkpoint that has the ability to suppress T cell immune activity. However, this pathway inhibits the immune activity of tumor-specific CD8^+^ T cells, allowing tumor cells to escape T-cell–mediated tumor-specific immunity, thereby promoting tumor development. Various studies have reported an association between increased expression of PD-L1 and poor prognosis in several cancers, including melanoma, lung cancer, ovarian cancer, and DLBCL (5–8). PD-L1 is also expressed on DLBCL tumor cells and tumor infiltrating nonmalignant cells, primarily macrophages (9, 10). Furthermore, the presence of high levels of PD-L1 is associated with poor OS and acts as a potent novel biomarker in DLBCL (7). Extensively pretreated patients with relapsed or refractory DLBCL achieve beneficial therapeutic effects with blockade of the PD-1/PD-L1 pathway (11–13). These results suggest that the PD-1/PD-L1 pathway contributes to tumor cell survival and that manipulation of this pathway may be an applicable therapeutic modality to treat DLBCL.

The tumor microenvironment(TME) of B-cell lymphoma significantly contributes to tumor progression and immune evasion (14). Recently, some researchers separate tumor immune microenvironment(TIME) from the TME, focusing on the immune cells around tumor cells (15, 16). The TIME in B cell malignancies includes multiple different cell types, including NLCs/tumor-associated macrophages(TAMs), tumor-infiltrating lymphocytes(TILs), regulatory T cells, dendritic cells(DCs), myeloid suppressor cells(MSCs) and endothelial stromal cells, which interact with and are enlisted by malignant cells (17, 18). Research has revealed that in DLBCL, the microenvironment is crucial for the provision of survival and proliferation signals and makes critical contributions to both disease progression and drug resistance/disease relapse (14). However, the status of the TIME and checkpoint molecules in A-DLBCL remains unclear. The aim of this study was to evaluate the checkpoint molecules and TIME of A-DLBCL and their associations with clinicopathological features and prognosis.

## Materials and Methods

### Patients and Samples

We analyzed 30 patients with A-DLBCL and 50 patients with ordinary DLBCL, both originally diagnosed, from June 2004 to April 2016. Four pathologists(M.L., Q.Y., S.G. and Z.W.) reviewed all cases according to the 2016World Health Organization classification of tumors of hematopoietic and lymphoid tissues (2). All cases were tested by EBER hybridization, and EBV-positive cases were excluded. The diagnosis was based on lymph node biopsy specimens of 20 patients or from the resection samples of extranodal involvement in the other 10 patients. The corresponding medical records were reviewed to obtain clinical information. A total of 30 well-documented patients were treated with different chemotherapy regimens, including cyclophosphamide, doxorubicin, vincristine, and prednisone(CHOP) or rituximab plus cyclophosphamide, doxorubicin, vincristine, and prednisone(R-CHOP), or etoposide, prednisone, vincristine, cyclophosphamide and doxorubicin(EPOCH). All 30 cases were exactly came from our previous 35 A-DLBCL study (3), while in this study we deleted 5 cases with incomplete data vital to analyze. **Table 1** summarizes the major clinical characteristics, treatment, and follow-up for all 30 patients. These patients and their tumors were compared with 50 cases of DLBCL-NOS without anaplastic features (common DLBCL). The 50 consecutive control cases were derived from one institution (Xijing Hospital) spanning the time interval of April 2009 through September 2014, and only DLBCL-NOS cases were enrolled in the study. Institutional ethical approval was obtained in compliance with the Helsinki Declaration.

**Table 1:** Clinical Features of the 30 Patients With ADLBCL.

Characteristics	Values (n[%])
Age (y)	
Median	61.5
Range	26-89
Sex	
Male	20(66.7)
Female	10(33.3)
Stage at diagnosis (Ann Arbor stage)	
I	1(3.3)
II	3(10)
III	8(26.7)
IV	13(43.3)
B symptoms	11(36.7)
Extranodal sites ≥ 2	12(40)
Serum LDH	
Normal	9(30)
High	16(53.3)
Chemotherapy CR rate	6(15)
IPI score	
1	2(6.7)
2	7(23.3)
3	8(26.7)
4	4(13.3)
5	4(13.3)
Immunophenotype	
Non-GCB subtype	25(83.3)
BCL-2	21(70)
c-MYC	17(56.7)
MYC/BCL2 DEL	13(21.7)
Ki-67( ≥ 80%)	20(66.7)
p53	24(80)
Fluorescence in situ hybridization	
MYC abnormalities	10(33.3)
BCL2 abnormalities	10(33.3)
BCL6 abnormalities	11(36.6)
Concurrent abnormalities of MYC and BCL2 and/or BCL6	9(30)
Mutation statuses	
TP53 MUT	17(56.7)
DEL, double-expressor lymphoma	

### Immunohistochemistry and Fluorescence *In Situ* Hybridization (FISH) Analysis

All immunohistochemical staining was performed using fully automated protocols on a Bond-III Autostainer (Leica Biosystems, Melbourne, Australia). Sections were subjected to staining protocols (detailed information on the antibodies is listed in **Table S1**).

The tumor immune microenvironment was characterized by immunostaining for CD3(tumor-infiltrating lymphocytes, TILs), CD8(cytotoxic T lymphocytes), T-bet(Th1 cells), GATA3(Th2 cells), FOXP3(regulatory T cells), CD68(tumor-associated macrophages, TAMs), CD163(M2-TAMs), and CD33(myeloid-derived suppressor cells, MDSCs). Germinal center B cell(GCB) and non-GCB subtypes of DLBCL were classified using the Hans (19) and Choi (20) algorithms. Each marker was investigated in a single stain with 5 high-power fields (HPFs) of the representative areas evaluated (21). PD-L1/PAX5 IHC double staining was used to evaluate PD-L1 expression in tumor cells (PD-L1^+^) or in nonmalignant stromal cells (defined as microenvironmental PD-L1, mPD-L1^+^). PD-L1 staining on tumor cells was considered positive in cases with moderate (2+) or strong (3+) cytoplasm reaction, and the percentage of the positive tumor cells was set as above 30%. Once reaching the 30% threshold, the PD-L1 positivity of nonmalignant stromal cells was ignored. Among PD-L1-negative DLBCL cases, in which PD-L1^+^nonmalignant microenvironment cells represented 20% or more of the total tissue, cellularity was defined as microenvironmental PD-L1(mPD-L1^+^) DLBCL (22). PD-1^+^ TILs were similarly evaluated. The number of tumor cells and cells in the TME were quantified in whole-tissue sections of all samples using an automated scanning microscope image analysis system (Ariol 2.1, SL-50; Applied Imaging, Melville, NY). Quantification of cells was performed with the nuclear Kisight assay provided by the manufacturer (Applied Imaging) (23).

FISH analysis was performed using LSI probes for PD-L1(Vysis, Abbott Laboratories, USA) on a Thermobrite System (Abbott Laboratories S500-24, USA) according to the manufacturer’s instructions. Images were collected using a workstation equipped with software (Imstar Pathfinder CellscaFluoSpot, France). Areas with a minimum of 70% tumor cells were counted, and signals from 100 non overlapping nuclei were analyzed. Positivity was determined above and a 30% threshold for extra copies (defined as copy number ≥ 3/cell). The 9p24.1 gene (PD-L1 gene) was labeled with Spectrum Red, and centromeres were labeled with Spectrum Green as a control probe on 9p. After calculation of red and green in areas with a minimum of 70% tumor cells were counted, and signals from 100 non-overlapping nuclei, the numbers of red and green was calculated. 4 or more was considered as amplification. 3 was considered as copy gains (24).

### Statistical Analysis

Statistical analysis including data description was performed using the Statistical Package of Social Sciences 14.0 software (Chicago, IL, USA). Pearson’s χ^2^ statistic, Fisher’s exact test or Spearman’s correlation test was used to analyze relationships between the markers and the clinical variables. The Kaplan–Meier method was used for survival analyses. Two-sided P-values of <0.05 were considered to be statistically significant for all analyses.

## Results

### Patient Characteristics and Histologic Findings

Thirty patients with A-DLBCL were enrolled, of whom 20 were male (66.7%) and 10 were female (33.3%), with a median age of 61.5 years. Only 1(3.3%) patient was classified as Ann Arbor stage I, 3(10%) as Ann Arbor stage II and 8(26.7%) as Ann Arbor stage III. The remaining 13(43.3%) patients were at Ann Arbor stage IV. Sixteen (53.3%) patients had elevated serum LDH, while 9(30%) had normal LDH levels. Clinical data are summarized in **Table 1**.

### Immunohistochemical and FISH Analysis of PD-L1 in Tumor Cells

**Figure 1:**
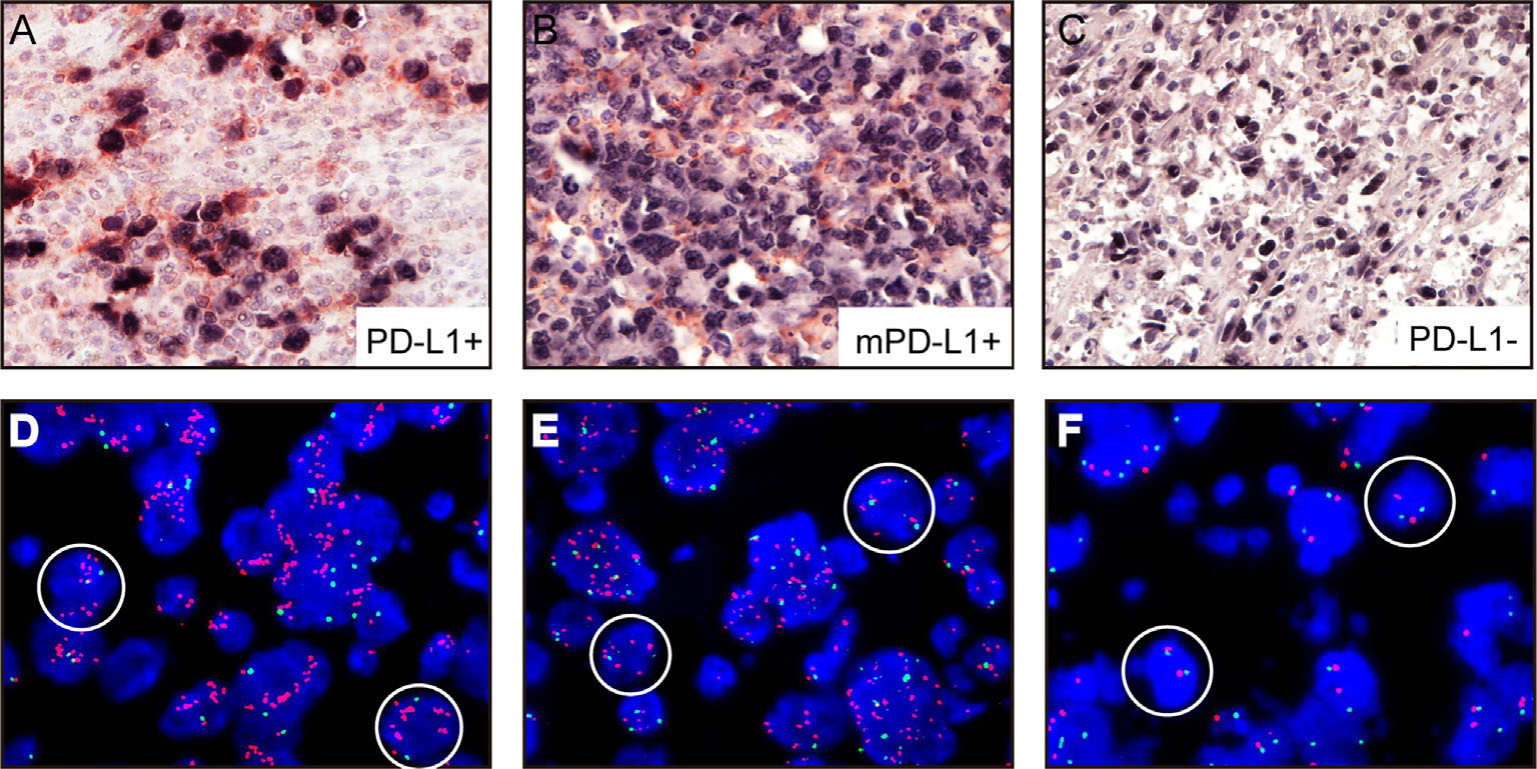
Representative immunohistochemical analysis of PD-L1 and PD-L1 FISH in A-DLBCL (400×magnification). **(A)** PD-L1^+^ A-DLBCL, tumor cells were double positive for PD-L1 (red) and PAX5 (black); **(B)** mPD-L1^+^ DLBCL, tumor cells were positive for PAX5 and negative for PD-L1; **(C)** PD-L1- A-DLBCL, tumor cells were negative for PD-L1 (red) and positive for PAX5 (black); **(D)** Copy number gains in PD-L1 locus; **(E)** Amplification in PD-L1 locus; **(F)** Normal PD-L1 FISH signal.

All A-DLBCL cases contained large bizarre-shaped tumor cells with abundant cytoplasm containing irregularly shaped nuclei. Membrane expression of PD-L1 by the tumor cells was observed in the investigated sections. PD-L1^+^ A-DLBCL was identified based on double immunostaining for PD-L1 and PAX-5. Representative IHC images for PD-L1 are shown in **Figures 1A–C**. The prevalence of PD-L1^+^A-DLBCL was 40% (12 of 30), which was higher than that of ordinary DLBCL (10%, P=0.001) according to our results.

Amplifications targeting the PD-L1 locus were observed in 7 out of 30 A-DLBCL cases. Seven cases (23.3%) were amplifications, and 2(6.6%) were copy gains, and the other 21 cases (70%) were normal. Representative examples of cytogenetic alterations identified by FISH are presented in **Figures 1D–F**. Detailed features of 7 A-DLBCL cases with PD-L1 locus gain were listed in **Supplementary Table 2**. In 50 ordinary DLBCL cases, only 2 cases(4%) harbored amplifications and 2 cases (4%) were copy gains. In comparison with ordinary DLBCL cases, extra copies of PD-L1 were more frequent in A-DLBCL (23.3% *vs* 4.0%, P=0.001).

### Composition and Distribution of the TIME

**Figure 2:**
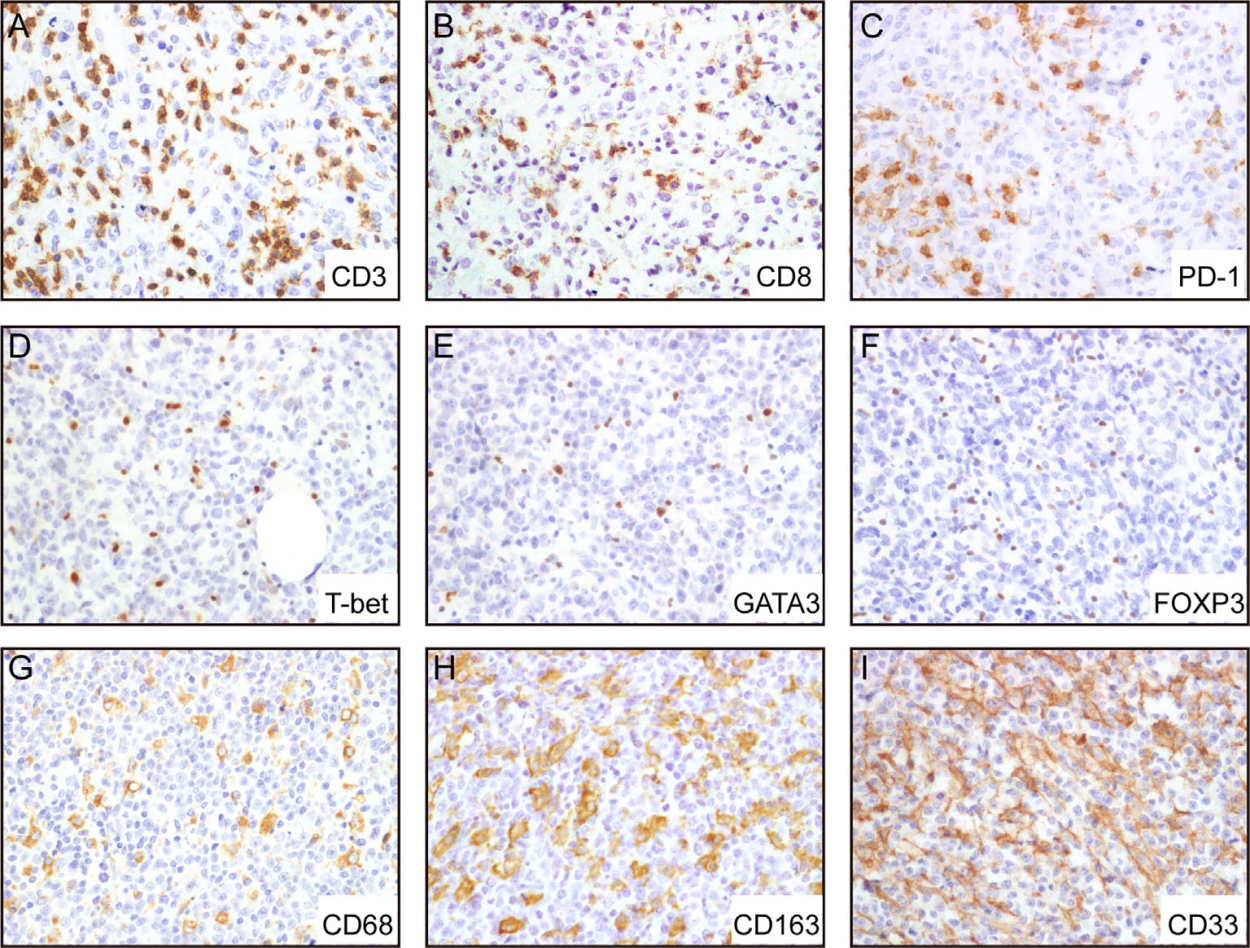
Representative immunohistochemical analysis of A-DLCBCL. Representative IHC staining expressions of CD3 **(A)**, CD8 **(B)**, PD-1 **(C)**, CD68 **(G)**, CD163 **(H)**, CD33 **(I)**, and negative expressions of T-bet **(D)**, GATA3 **(E)**, FOXP3 **(F)**.

To evaluate the cellular composition of the TIME in A-DLBCL, we used markers targeting various cells of the TIME in IHC analysis. Representative immunostaining of A-DLBCL cases is shown in **Figure 2**. The immunohistochemical and FISH results of 30 Patients with A-DLBCL are listed in **Table 2**. Afterwards, the differences in TIME in A-DLBCL and ordinary DLBCL cases were analyzed and are listed in **Table 3**.

**Table 2:** Immunohistochemical and FISH Analysis of 30 Patients with A-DLBCL.

Patient Number	PD-L1			TIME								
	Tum	mPD-LI^**^	PD-L1	PD-1 TIL	CD3	CD8	T-bet	GATA3	FOXP3	CD68	CD163	CD33
	PD-L1*		FISH***	(/HPF****)	(/HPF)	(/HPF)	(/HPF)	(/HPF)	(/HPF)	(/HPF)	(/HPF)	(/HPF)
1	+		+	10	70	20	55	0	0	80	70	20
2	−	−	−	50	130	55	60	15	0	100	80	15
3	+		+	0	160	140	120	110	120	90	50	50
4	−	+	−	100	150	30	60	30	0	80	60	20
5	+		+	0	110	60	75	15	0	60	50	40
6	+		−	0	50	35	45	5	0	70	50	50
7	−	+	−	0	130	70	110	120	100	60	60	40
8	−	−	−	60	150	40	120	5	10	85	80	0
9	+		+	0	150	30	130	0	0	110	90	0
10	−	+	−	0	80	10	60	20	0	70	60	25
11	−	+	−	0	50	2	10	60	0	50	50	20
12	−	−	−	0	50	5	0	30	15	25	0	0
13	−	−	−	20	50	50	0	0	0	60	50	30
14	+		−	30	160	30	100	80	25	70	60	5
15	+		+	120	150	35	80	30	15	80	70	80
16	+		−	0	5	5	0	0	0	50	80	10
17	−	+	−	70	150	40	50	100	50	50	40	30
18	−	+	−	70	140	120	30	10	10	80	80	60
19	−	−	−	10	120	35	90	95	90	90	80	60
20	−	+	−	0	90	10	0	0	0	65	20	30
21	+		−	25	100	20	60	40	60	50	40	25
22	+		−	0	80	40	0	0	0	40	80	60
23	−	+	−	20	40	35	3	0	0	60	45	50
24	+		+	0	60	40	20	30	0	30	5	80
25	−	+	−	5	120	3	50	20	10	50	50	25
26	−	+	−	10	50	25	15	0	0	65	40	30
27	−	+	−	0	40	20	10	5	0	85	80	70
28	−	+	−	0	70	5	55	0	0	60	60	50
29	−	+	−	40	30	10	0	0	0	60	60	10
30	+		+	60	120	20	30	80	20	40	40	30

*Tum PD-L1, tumor PD-L1.

**mPD-L1, microenvironment PD-L1. Once tumor PD-L1 was defined positive, the mPD-L1 positivity was ignored.

***“+” stands for amplification of PD-L1 in tumor cells.

****/HPF, per high power field.

**Table 3:** Comparison of TME markers and checkpoint molecules between A-DLBCL and ordinary DLBCL.

Features	A-DLBCL (n = 30)	Ordinary DLBCL (n = 50)	P value
PD-L1+	12/30 (40%)	5/50 (10%)	0.001
mPD-L1+	13/18 (72.2%)	15/45 (33.3%)	0.005
CD3+	78.2 ± 7.2/HPF	92.5 ± 8.6/HPF	0.214
CD8+	34.7 ± 5.7/HPF	58.6 ± 7.5/HPF	0.026
PD-1+ TILs	23.3 ± 6.0/HPF	50.6 ± 7.2/HPF	0.01
T-bet+	49.8 ± 7.6/HPF	41.1 ± 6.3/HPF	0.394
GATA3+	30.5 ± 7.1/HPF	15.1 ± 3.8/HPF	0.039
FOXP3+	17.5 ± 6.0/HPF	6.6 ± 2.1/HPF	0.048
CD68+	65.5 ± 3.6/HPF	59.9 ± 5.5/HPF	0.461
CD163+	56 ± 4.0/HPF	50.3 ± 6.3/HPF	0.514
CD33+	33.8 ± 4.2/HPF	20.6 ± 4.1/HPF	0.039

#### mPD-L1 Expression

In addition to cases containing PD-L1^+^ large bizarre-shaped tumor cells, there were also cases with PD-L1^+^ nonmalignant stromal cells, and we defined them as mPD-L1^+^ (PD-L1^+^/PAX5^-^) (**Figure 1B**). PD-L1/PAX5 double staining showed that the proportion of mPD-L1^+^ cells was higher (72.2% *vs* 33.3%, P=0.005) in A-DLBCL patients than patients with ordinary DLBCL.

#### MDSCs and Tumor-associated Macrophages

We found that MDSCs were more abundant in A-DLBCL than in ordinary DLBCL(P=0.039), with 33.8 ± 4.2/HPF *vs* 20.6 ± 4.1/HPF, represented by CD33, while M2 TAMs (CD163) and M1/M2 TAMs (CD68) showed no significant difference between A-DLBCL and ordinary DLBCL.

#### TILs

The distribution of TILs was diffuse in all cases. The difference between the numbers of cells expressing GATA3 and FOXP3 in A-DLBCL and DLBCL was significant(P=0.039; P=0.048) after statistical analysis, indicating that there were more Th2 cells and Treg cells in the TME of A-DLBCL than in ordinary DLBCL. In contrast, staining of CD8 showed decreased numbers of CD8^+^ T cells (34.7 ± 5.7/HPF *vs* 58.6 ± 7.5/HPF, P=0.026) and PD-1^+^ TILs (23.3 ± 6.0/HPF *vs* 50.6 ± 7.2/HPF, P=0.010) in A-DLBCL compared to ordinary DLBCL. Moreover, there seemed to be no significant difference in CD3^+^ T cells and Th1 cells between A-DLBCL and ordinary DLBCL cases.

### Clinicopathological Association and Prognostic Factors

**Figure 3:**
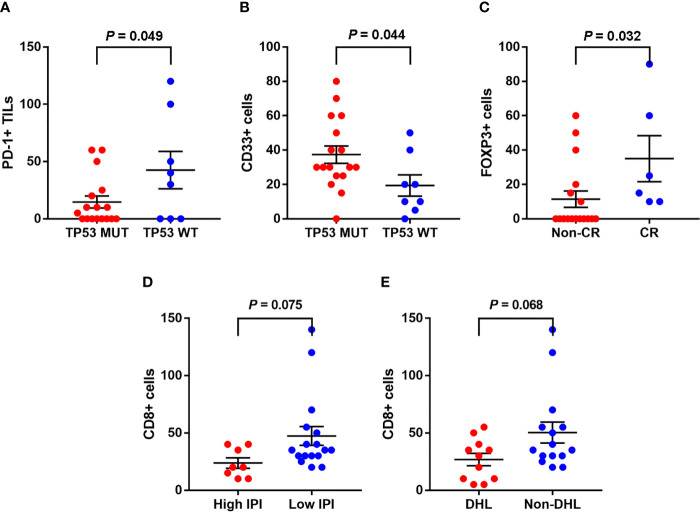
Associations between clinicopathological features and the tumor immune microenvironment markers in A-DLBCL. There were less PD-L1^+^ TILs in TP53 mutant cases than in TP53 wild type cases **(A)**, the result was opposite as to CD33^+^ cells (MDSCs) **(B)**. Patients with less Treg cells (10.3 ± 4.5/HPF vs 35 ± 13.4/HPF, P=0.032) in TME (FOXP3) ended in non-complete response (CR) to chemotherapy **(C)**. CD8^+^ cells showed a trend of decreasing in number in patients with high International Prognostic Index (IPI) score **(D)**. CD8^+^ cells showed a trend of decreasing in patients with double-hit lymphoma (DHL) **(E)**.

Same as in our previous study (3), the mutation states of ADLBCL cases were already performed and all were available in cases. In this study, TP53 mutation status was associated with the numbers of PD-L1^+^ TILs (CD3) and MDSCs (CD33) in A-DLBCL cases. There seemed to be fewer PD-L1^+^ TILs in TP53 mutant cases than in TP53 wild type cases (14.7 ± 5.2/HPF *vs* 42.5 ± 16.3/HPF, P=0.049). For MDSCs, the result was the opposite (37.4 ± 5.0/HPF *vs* 19.3 ± 6.2/HPF, P=0.044). The comparison also showed that patients with fewer Treg cells (10.3 ± 4.5/HPF *vs* 35 ± 13.4/HPF, P=0.032) in the TIME(FOXP3) had a non-complete response(Non-CR) to chemotherapy, since high FOXP3 expression had a significant correlation with complete response. The number of CD8^+^ cells showed a decreasing trend in patients with high International Prognostic Index (IPI) scores (23.8 ± 5.7/HPF *vs* 47.4 ± 8.2/HPF, P=0.075). All results are shown in **Figure 3**.

**Figure 4:**
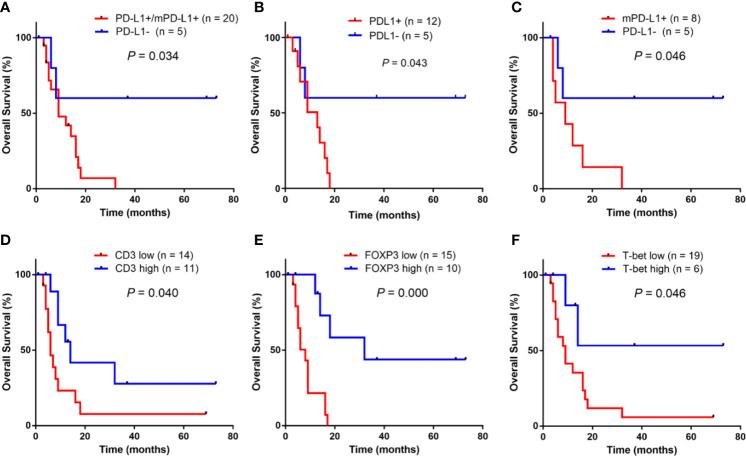
Univariate survival analysis for anaplastic variant of diffuse large B-cell lymphoma In ADLBCL, patients with PD-L1^+^ and/or mPD-L1^+^ **(A–C)** had a significantly poorer overall survival (OS) than those with PD-L1^-^.An increase in CD3^+^ cell numbers, FOXP3^+^ cells numbers and T-bet^+^ cell numbers were significantly associated with prolonged OS in patients with A-DLBCL was significantly worse than those who tested negative (*P* < 0.05) **(C–F)**.

**Figure 4** shows the univariate overall survival analysis of A-DLBCL patients according to the expression of different markers, showing that patients with PD-L1^+^/mPD-L1^+^ or mPD-L1^+^ had significantly poorer overall survival(OS) than those with PD-L1^-^ status(P=0.034 and P=0.046, respectively). In consistent with DLCBL, A-DLBCL patients with PD-L1^+^ has a poorer prognosis than PD-L1^-^. An increase in CD3^+^, FOXP3^+^ and T-bet^+^ cell numbers was significantly associated with prolonged OS in patients with A-DLBCL (P=0.040, P=0.000 and P=0.046, respectively).

## Discussion

In this study, we demonstrated the molecular pathogenesis of primary A-DLBCL and its association with clinical characteristics. In brief, our findings showed high expression of PD-L1^+^ and mPD-L1^+^ in A-DLBCL compared with ordinary DLBCL. In the TME, the numbers of PD-1^+^ TILs and CD8^+^ cells were significantly lower in A-DLBCL. In contrast, the numbers of GATA3^+^ cells, FOXP3^+^ cells and CD33^+^ cells were significantly higher in A-DLBCL patients. These differences between A-DLBCL and ordinary DLBCL were associated with poor prognosis in A-DLBCL.

Traverse-Glehen et al. (25) divided GZL into 3 distinct subgroups (namely, cHL-like GZL, large B-cell lymphoma-like GZL, and composite/sequential cases) according to the evaluation and integration of tumor cell morphology, architecture and growth pattern, microenvironmental composition, and immunophenotype. CHL-like GZL and LBCL-like GZL were then divided into 2 subgroups (namely, groups 0 and 1 for cHL-like cases and groups 2 and 3 for LBCL-like cases).A-DLBCL differs from these four subtypes as we stained for CD15 with immunohistochemistry. In CD15 IHC found that it was negative in all of our cases, and A-DLBCL cases showed a large B-cell-like morphology with an obvious sinusoidal growth pattern and was not on an inflammatory background.

PD-1 belongs to the B7/CD28 family and is expressed on the surface of activated T and B cells, follicular helper T cells, dendritic cells (DCs), and monocytes. The interaction between PD-1 and its ligand PD-L1 reduces T cell proliferation and cytokine release and inhibits survival proteins (e.g. bcl-xl), which results in apoptosis (21, 26, 27). Non-Hodgkin lymphomas harbor decreased PD-L1 expression with the notable exceptions of nodal diffuse large B-cell lymphoma(DLBCL) and virus-associated lymphoma (28). Retrospective analyses have shown that PD-L1 positivity in tumor cells, as detected by immunohistochemistry, may predict an improved response to anti-PD-1/PD-L1 therapy in melanoma (29), non-small-cell lung carcinoma (30), breast cancer (31), esophageal adenocarcinoma (32), glioblastoma (33), renal cell carcinoma (34), pancreatic carcinoma (35) and urothelial carcinoma (36). Consistent with Kiyasu et al. in DLBCL patients, our findings on A-DLBCL patients also revealed that PD-L1 expression was associated with poor overall survival. In the meantime, we confirmed the consistency of the cases of PD-L1 amplification and PD-L1 overexpression, which suggested that such cytogenetic alteration correlated with increased expression of PD-L1. Overall, these findings indicated that anti-PD-1/PD-L1 therapy could be a novel therapeutic approach for A-DLBCL.mPD-L1^+^ DLBCL is defined as 20% or more PD-L1^+^ nonmalignant cells among the total tissue cellularity in PD-L1^–^ DLBCL. Studies have shown that the prevalence of mPD-L1^+^ DLBCL is approximately 15% in DLBCL (22, 37). Our results told the same story as those DLBCL studies. In addition, patients with PD-L1^+^ and/or mPD-L1^+^ had significantly poorer overall survival (OS) than those with PD-L1^-^in DLBCL. These results may help explain both tumor escape from host immune surveillance and inhibition of activated T-cells in A-DLBCL.

TIME cells include tumor-infiltrating lymphocytes (TILs), regulatory T cells, and myeloid-derived suppressor cells (MDSCs). Some studies have demonstrated that PD-L1-expressing tumor cells can induce apoptosis in PD-1^+^ tumor-infiltrating lymphocytes (TILs). Others have reported that the quantity of PD-1^+^ TILs is significantly positively correlated not only with PD-L1 expression in tumor cells but also with PD-L1 expression in tumor cells/macrophages. Moreover, the therapeutic response can be affected by several parameters, such as the mutational load of GATA3^+^ cells, FOXP3^+^ cells and CD33^+^ cells and the abundance of TILs in lung cancer or melanoma (38–40). The TP53 gene is mutated in approximately 20% of cases of DLBCL; TP53 mutation predicts poor prognosis (41) and is widely used in clinical evaluation. Patients with more CD3^+^cells have lower risks of relapse after antitumor treatment. In speaking of FOXP3, the prognostic influence is controversial, reported as being correlated with a good prognosis in some studies or with an adverse outcome in others (42–44). To be detailed, in follicular lymphoma, germinal center-like diffuse large B-cell lymphoma and classical Hodgkin’s lymphoma, the increased FOXP3+ Treg cells being associated with better OS, while in non-germinal center diffuse large B-cell lymphomas the conclusion is opposite. As we observed a significantly reduced quantity of PD-1^+^ TILs, an increased quantity of CD33^+^ cells, and a high possibility of non-CR to treatment related to a low quantity of FOXP3^+^ cells in our study, we propose that the prognosis of A-DLBCL is poorer than that of ordinary DLBCL. Accumulating evidence suggests that myeloid-derived suppressor cells(MDSCs) (45, 46) can enhance their inhibitory function to further dampen the T cell mediated antitumor response. In accordance with the observations of Y. Liu et al., our findings may suggest that the antitumor response is inhibited within the malignant node. Although some studies point towards a negative prognostic impact of regulatory T cells in lymphoma (47, 48), it is possible that regulatory T cells directly suppress malignant B cells or counteract tumor-supporting T cells. Moreover, the abundance of Tregs has been proven by research on predictors of progression-free survival (49). In our study, CD3 and FOXP3 were related to opposite out comes, which deserves further study.

Several studies have used CD163 as an M2 tumor-associated macrophage (TAM) marker and CD68 as a pan macrophage marker(M1+M2) (50–52). Emerging research has shown that M2-like TAMs expressing PD-L1 correspond to the majority of immune cells in DLBCL and may play a role in tumor immune escape, angiogenesis, or matrix remodeling; in addition, a high M2-TAM level at diagnosis may be an unfavorable prognostic factor in DLBCL patients (53). Unfortunately, our data did not show results consistent with those of previous DLBCL studies, and there was no significant difference in M1 and M2 cell quantity between A-DLBCL and ordinary DLBCL(P=0.461 and 0.514), which may suggest a less important role for TAMs in A-DLBCL.

T-bet and GATA3 are two transcription factors that determine Th cell differentiation into Th1 or Th2 cells, respectively, and both are expressed in the nuclei of tumor-infiltrating lymphoid cells. Immune balance controlled by Th1 and Th2 cells is critical for protecting the host against pathogenic invasion, while imbalance can cause various immune disorders. We found that the expression of Th1 markers did not show a significant difference in A-DLBCL and ordinary DLBCL (P=0.394), while the expression of Th2 markers in A-DLBCL was increased. According to existing reports, increased Th2 marker levels accelerate the secretion of interleukin(IL)-10 and IL-4 and inhibit cellular immune function, and the GATA3/T-bet(G/T) ratio of tumor-infiltrating lymphoid cells is an independent negative predictive marker for survival (54, 55). Overall, our findings verify the elevated G/T ratio in A-DLBCL, in line with studies of gene-expressing profiles in nodal DLBCL, revealing a high number of M2 macrophages in the TIME (56). All of the above findings suggest that there may be an immunosuppressive state in/around the focus in A-DLBCL patients. Moreover, this could be one of the potential mechanisms that explains why the prognosis of A-DLBCL is worse than that of ordinary DLBCL. CD8^+^ T cells are generally thought to play a central role in the antitumor immune response, and the presence of CD8^+^has been reported as a prognostic factor in cancer (57, 58). Our results showed that a significantly lower level(P=0.026) of CD8^+^ T cells existed in A-DLBCL, suggesting an immunosuppressive state within the focus.

The standard therapy for patients with DLBCL contains rituximab, cyclophosphamide, doxorubicin, vincristine, and prednisone(R-CHOP). By such regimen, approximately 60–70% of patients with DLBCL are cured of the disease. However, 30–40% of patients experience disease relapses or, in a small patient subset, are refractory to R-CHOP therapy. The great potential of our recent advances is based on the understanding of the molecular characteristics of DLBCL, and we hope this will be translated into the development of novel, highly effective therapies for patients with A-DLBCL. With the advent of novel targeting agents/regimens, one that specifically targets A-DLBCL will eventually be developed. In addition, our final cohort contains only 30 A-DLBCL cases, which make the results relatively preliminary. Our study requires more cases to make the conclusion closer to the real world.

## Conclusion

In conclusion, an immunosuppressive state in A-DLBCL may be described by both high levels of PD-L1^+^, mPD-L1^+^, CD33^+^, CD163^+^ and GATA3^+^ cells and low levels of CD8^+^ and PD-1^+^cells in the TIME; these TIME features could highlight key molecular markers of the prognosis of A-DLBCL, which is poorer than that of ordinary DLBCL. A-DLBCL displays a distinct pattern of tumor immune microenvironment components and checkpoint molecules that distinguish it from ordinary DLBCL. These results could help in understanding the prognosis of A-DLBCL patients and determining therapeutic strategies that target the tumor immune microenvironment.

## Data Availability Statement

The original contributions presented in the study are included in the article/**Supplementary Material**. Further inquiries can be directed to the corresponding authors.

## Ethics Statement

The studies involving human participants were reviewed and approved by Medical Ethics Committee of the First Affiliated Hospital of the Air Force Medical University (Forth Millitary Medical University). The patients/participants provided their written informed consent to participate in this study.

## Author Contributions

RL, ML and ZW contributed to the conception of the study; JX and KY performed the experiment; DZ, JM, JC, KW and YL contributed significantly to analysis and manuscript preparation; TX, JC and KW performed the data analyses and wrote the manuscript; QJ contributed greatly to the revised manuscript. YW, LF, QY, SG, GC, QC, HX, FL and CQ helped perform the analysis with constructive discussions. All authors contributed to the article and approved the submitted version.

## Funding

National Natural Science Foundation of China (81570180).

## Conflict of Interest

The authors declare that the research was conducted in the absence of any commercial or financial relationships that could be construed as a potential conflict of interest.

## Supplementary Material

Supplementary Table 1 Detailed Information of Primary Antibodies.

Supplementary Table 2 Detailed Features of A-DLBCL Cases with PD-L1 Gain.
