# Supplementary material for: Tumor Immune Microenvironment Components and Checkpoint Molecules in Anaplastic Variant of Diffuse Large B-Cell Lymphoma
Source: Front Oncol. 2021 Jun 16;11:638154. doi: 10.3389/fonc.2021.638154 (PMC8242181; doi:10.3389/fonc.2021.638154)
Supplement: Supplementary Table 1 — Detailed Information of Primary Antibodies. [file Table_1.pdf]

Supplementary Table 1. Detailed Information of Primary Antibodies

Antibody	Source	Clone	Dilution
PD-L1	MXB Biotech	MXR003	Ready-to-use
CD3	MXB Biotech	SP7	Ready-to-use
CD8	MXB Biotech	SP16	Ready-to-use
T-bet	Abcam	4B10	1:1000
GATA3	Abcam	EPR16651	1:500
FOXP3	Abcam	EPR22102-37	1:500
CD68	MXB Biotech	KP1	Ready-to-use
CD163	MXB Biotech	10D6	Ready-to-use
CD33	Abcam	EPR23051-101	1:200
