# Supplementary material for: Tumor Immune Microenvironment Components and Checkpoint Molecules in Anaplastic Variant of Diffuse Large B-Cell Lymphoma
Source: Front Oncol. 2021 Jun 16;11:638154. doi: 10.3389/fonc.2021.638154 (PMC8242181; doi:10.3389/fonc.2021.638154)
Supplement: Supplementary Table 2 — Detailed Features of A-DLBCL Cases with PD-L1 Gain. [file Table_2.pdf]

TABLE S2. Detailed Features of A-DLBCL Cases with PD-L1 Gain

Features	Patient Number*						
	**1**	**3**	**5**	**9**	**15**	**24**	**30**
Age (y)	64	67	75	72	68	46	47
Sex	Male	Male	Female	Male	Male	Female	Male
Stage at diagnosis (Ann Arbor stage)	IV	IV	IV	IV	IV	III	IV
B symptoms	Y	Y	Y	N	Y	N	Y
Numbers of extranodal sites	2	3	2	4	4	3	3
Serum LDH	High	High	High	High	Normal	Normal	High
Chemotherapy Effect	PD	PD	PD	PD	PR	PD	PR
IPI score	5	5	3	5	3	3	5
GCB or Non-GCB							
Hans	Non-GCB	Non-GCB	Non-GCB	Non-GCB	Non-GCB	Non-GCB	Non-GCB
Choi	Non-GCB	Non-GCB	Non-GCB	Non-GCB	Non-GCB	GCB	Non-GCB
Morphology	Scattered	Scattered	Scattered	Numerous	Numerous	Scattered	Scattered
Immunophenotype							
CD5	-	-	-	-	-	-	-
CD30	+	+	-	+	-	+	-
c-MYC	-	+	+	+	-	-	+
BCL-2	+	+	+	+	-	+	-
Ki-67	80%	90%	80%	90%	70%	50%	80%
p53	Variable ＞50%	Diffuse	Diffuse	Variable ＜50%	Variable ＜50%	Diffuse	Variable ＞50%

* Patient numbers correspond to those in **Table 2**
